# Dimeric Histidine as a Novel Free Radical Scavenger Alleviates Non-Alcoholic Liver Injury

**DOI:** 10.3390/antiox10101529

**Published:** 2021-09-27

**Authors:** Zizhen Zhao, Chen Fu, Yuping Zhang, Ailing Fu

**Affiliations:** School of Pharmaceutical Sciences, Southwest University, Chongqing 400716, China; Zhaozizhen@swu.edu.cn (Z.Z.); Fuchen0794@swu.edu.cn (C.F.); Zyp19980101@swu.edu.cn (Y.Z.)

**Keywords:** nonalcoholic liver injury, H-bihistidine, fatty liver, liver fibrosis, oxidation injury

## Abstract

Non-alcoholic liver injury (NLI) is a common disease worldwide. Since free radical damage in the liver is a crucial initiator leading to diseases, scavenging excess free radicals has become an essential therapeutic strategy. To enhance the antioxidant capacity of histidine, we synthesized a protonated dimeric histidine, H-bihistidine, and investigated its anti-free radical potential in several free-radical-induced NLI. Results showed that H-bihistidine could strongly scavenge free radicals caused by H_2_O_2_, fatty acid, and CCl_4_, respectively, and recover cell viability in cultured hepatocytes. In the animal model of nonalcoholic fatty liver injury caused by high-fat diet, H-bihistidine reduced the contents of transaminases and lipids in serum, eliminated the liver’s fat accumulation, and decreased the oxidative damage. Moreover, H-bihistidine could rescue CCl_4_-induced liver injury and recover energy supply through scavenging free radicals. Moreover, liver fibrosis prepared by high-fat diet and CCl_4_ administration was significantly alleviated after H-bihistidine treatment. This study suggests a novel nonenzymatic free radical scavenger against NLI and, potentially, other free-radical-induced diseases.

## 1. Introduction

Liver disease is a significant source of global health problems. Nonalcoholic liver injury (NLI), including nonalcoholic fatty liver (NAFL), and drug- and poison-induced liver injury, is becoming a challenge to human health worldwide [1]. The NLI can gradually develop to nonalcoholic steatohepatitis, which leads to cirrhosis and hepatocellular carcinoma [2]. Despite the growing public health impact of NLI, therapeutic strategy is limited and there is still a lack of clinically approved therapies.

Excess free radical accumulation is an important cause of NLI [3]. In NAFL patients, abnormal lipid metabolism induces the imbalance of redox homeostasis and the increase in free radical levels in hepatocytes [4]. The free radicals can cause lipid peroxidation, and destroy the structure and function of protein and DNA, leading to membrane rupture and cell death. Therefore, improving hepatocyte anti-free radical capacity has been considered an effective strategy in treating the liver injury.

Recently, there has been a growing interest in amino acids with antioxidant activity that can prevent the deleterious effects of reactive oxygen species (ROS) in the liver [5]. It is known that histidine is a conditionally essential amino acid for mammals, and the previous report suggests that histidine supplementation can reduce the level of oxidative stress in obese women and obese rats caused by high-fat diets [6]. In addition, histidine administration is negatively correlated with 8-OHdG level (an oxidative stress biomarker) in epididymal sperm [7]. Nevertheless, the anti-free radical capacity of histidine is relatively weak in treating NLI, and it is hard to be used as an effective agent against liver injury in animals.

To enhance the anti-free radical capacity of histidine, we transform histidine into protonated dimer histidine (H-bihistidine), and then explore the anti-free radical effect of the compound in cultured hepatocytes and animal models of NLI by using several free radical inducers, including H_2_O_2_, fatty acid, and CCl_4_. The results show that H-bihistidine has a solid activity to eliminate various free radicals and restore cell viability. For the first time, this study suggests that the protonated amino acid would be a novel therapeutic candidate for treating NLI and potentially for other free-radical-induced injuries.

## 2. Materials and Methods

### 2.1. Synthesis of H-Bihistidine

All reagents were procured from Alfa, Aldrich (Miami, FL, USA), and further utilized without purification. In a 10 mL closed brown vial attached with a septum, a solution of H_2_O/MeCN (2 mL/2 mL) within l-histidine (2.0 g) was purged with N_2_ for 15 min, and then 4 equivalents of NH_4_Cl was mixed. Afterward, the solution was stirred at room temperature for about 2 h. The resultant suspension was dried in vacuum, and the powder was purified by water and Et_2_O subsequently. Then, the residue was dried in vacuum to obtain the purified product. The structure was analyzed by Fourier transform infrared spectroscopy (FTIR; Thermo Scientific Nicolet; Waltham, MA, USA), nuclear magnetic resonance spectroscopy (NMR; Bruker AVANCE NEO; Karlsruhe, Baden-Württemberg, Germany), and high-resolution mass spectra (HRMS; Finnigan LTQ-FT instrument; Waltham, MA, USA), respectively.

### 2.2. Detection of H_2_O_2_ Content

KMnO_4_ titration method was used to detect H_2_O_2_ content [8]. Briefly, l-histidine and H-bihistidine were, respectively, added into H_2_O_2_ samples of test tubes, and then H_2_SO_4_ (3 mol/L) was further added to the solution. The solution was titrated with 0.02 mol/L KMnO_4_ standard solution. The H_2_O_2_ content was calculated from the consumption volume of KMnO_4_. Six isolated experiments were performed for each concentration of l-histidine or H-bihistidine.

### 2.3. Animals

Healthy C57BL/6J mice (18~22 g) were utilized in the experiments. The mice were procured from Chongqing Medical University, China. The control animals were preserved in SPF laboratory with standard mouse chow and water, while the model mice of fatty liver were given a high-fat diet. All animal experiments were accomplished under the guideline approved by the Institutional Animal Care and Use Committee of Southwest University, China (IACUC-SWU #2020-0036).

### 2.4. Cell Culture

Primary hepatocytes of the mouse were separated and cultured under sterile conditions based on the previous reports [9,10]. The C57BL/6J mice were fasted overnight and euthanized with overdose of pentobarbital sodium. Then, Hank’s perfusate containing 0.1% type IV collagenase was perfused through the inferior vena cava. The liver tissue was carefully removed and shaken in Dulbecco’s modified Eagle’s medium (DMEM) to disintegrate the tissue to obtain cell suspension. The suspension was filtered through a 200 mesh, and then centrifuged at 1000 rpm for 2 min. The cells were resuspended in DMEM and the percentage of living cell was calculated by trypan blue. When the percentage reached 70%, the cells were cultured in DMEM supplemented with 10% fetal bovine serum (FBS), 100 μg/mL streptomycin, and 100 units/mL penicillin in an incubator (37 °C and 5% CO_2_). All cell culture reagents were procured from Gibco Co. (Thermo Fisher Scientific, Waltham, MA, USA).

### 2.5. Preparation of Damaged Cells by Free Radicals and Biochemical Assay

Three free radical inducers (H_2_O_2_, fatty acid, and CCl_4_) were respectively used to examine the effects of H-bihistidine against cell damage. For H_2_O_2_-induced cell injury, H_2_O_2_ (3 mM) was mixed into the cell culture media for 2 h incubation when the cells grew up to 80% confluency [11]. For fatty-acid-induced cell injury, palmitate (1 mM) in DMSO was added to the media for 24 h [12] and, for cell injury caused by CCl_4_, the CCl_4_ (10 mM) in DMSO was introduced into the culture media and incubated for 12 h [13]. Then, H-bihistidine was respectively put into the individual media. The cell viability was evaluated by using the CCK method. Concisely, the cultured cells in a 96-well plate were rinsed with PBS (pH 7.4), and the CCK solution was added to each well based on the manufacturer’s operating instructions (Beijing Beyotime Biotech. Co., Beijing, China). After 4 h incubation, the absorbance was assayed at 570 nm wavelength on a microplate reader (Bio-Rad, Hercules, CA, USA). The cell viability was identified through OD (sample-blank)/OD (control-blank) × 100%. 

DCFH-DA was used as a probe to detect intracellular ROS. The cells were washed by PBS (pH 7.4) 3 times after being treated with H-bihistidine; then, DCFH-DA solution (20 mM) from a commercial kit (Beijing Beyotime Biotech. Co., Beijing, China) was added into the fresh media with the final concentration of 50 μM for 1 h incubation. After the cells were washed with PBS, a fluorescence spectrophotometer (Hitachi, F-7000; Hitachi, Japan) was used to quantify the ROS level according to the manufacturer’s instructions. In addition, fluorescence was observed under a confocal microscope (Zeiss, LSM 700; Jena, Germany), with excitation wavelength 488 nm and emission wavelength 525 nm. Moreover, after the cells were treated with H-bihistidine, the levels of ATP, glutathione (GSH), malondialdehyde (MDA), and superoxide dismutase (SOD) activity were, respectively, determined by using commercial kits (Nanjing Jiancheng Biotech. Ltd. Co., Nanjing, China). A set of eight individual experiments were conducted for every assay.

### 2.6. NAFL Model Preparation and H-Bihistidine Administration

The C57BL/6J mice were classified randomly into four groups. The normal control mice were given standard chow (*n* = 10), and the mice in other groups were fed with a high-fat diet composed of 38% standard diet, 40% fat, 2% cholesterol, and 20% fructose daily. Eight weeks later, the mice in the H-bihistidine therapy group were injected intravenously with H-bihistidine (5 mg/kg) once daily for 7 consecutive days, and the mice in the histidine therapy group were intravenously administrated the equal grams of histidine. The mice model group received an equal volume of saline. After administration, all mice were fasted for about 12 h, and then euthanized using pentobarbital sodium. Serum and liver tissues of the mice were isolated for tissue assay and biochemical detections.

The activity of serum alanine aminotransferase (ALT) and aspartate aminotransferase (AST), and content of total cholesterol (TC) and total triglyceride (TG) were, respectively, calculated utilizing an automatic biochemistry analyzer (Lab compare, Bedford, NH, USA). Moreover, liver tissues stored in 10% buffered formalin were further sectioned to be stained in oil red and, meanwhile, the tissue homogenate was prepared by snap freezing in liquid nitrogen. Then, the levels of ROS, MDA, GSH, SOD, and ATP in the tissue homogenate were calculated utilizing commercial kits.

### 2.7. Preparation of CCl_4_-Induced Liver Injury Mice

The mouse model of CCl_4_-induced liver injury was produced by subcutaneous injection of 5 μL 20% CCl_4_ (olive oil as solvent) once every two days for 3 weeks [14]. The control was given olive oil only. After CCl_4_ treatment for 3 weeks, the CCl_4_-treated mice were classified into three groups randomly (*n* =  10 in each group). Mice in the H-bihistidine-treated group were intravenously injected with 5 mg/kg H-bihistidine once a day for 7 days. The mice in histidine group were given an equal quantity of histidine. The model mice were injected with an equal volume of saline solution. After H-bihistidine treatment, all mice were euthanized and blood was obtained to prepare serum. Then, serum levels of ALT and AST were evaluated by using the automatic biochemistry analyzer. The liver tissue sections were stained utilizing hematoxylin–eosin (HE) staining. In addition, the levels of ROS, MDA, GSH, SOD, and ATP in the tissue homogenate were calculated utilizing the commercial kits.

### 2.8. Preparation of Mouse Model of Liver Fibrosis

The mouse model of liver fibrosis was prepared according to the report [15]. The C57BL/6J mice were fed with a high-fat diet for 12 weeks and, meanwhile, were subcutaneously given 5 μL 20% CCl_4_ once every two days. Then, the mice received the H-bihistidine or histidine treatment for one week. After the mice were euthanized, liver tissue was separated for section and tissue homogenate. The sections were stained by Sirius red, and hydroxyproline content in the tissue homogenate was assayed utilizing the Jamall methods, as reported earlier.

### 2.9. Statistical Analysis

The data were demonstrated as mean ± SD, and the results were examined by one-way analysis of variance (ANOVA) method, followed by Tukey post hoc test. Significant differences were determined as *p* < 0.05, and highly significant as *p* < 0.01.

## 3. Results

### 3.1. Synthesis of the Dimeric Histidine (H-Bihistidine)

**Figure 1:**
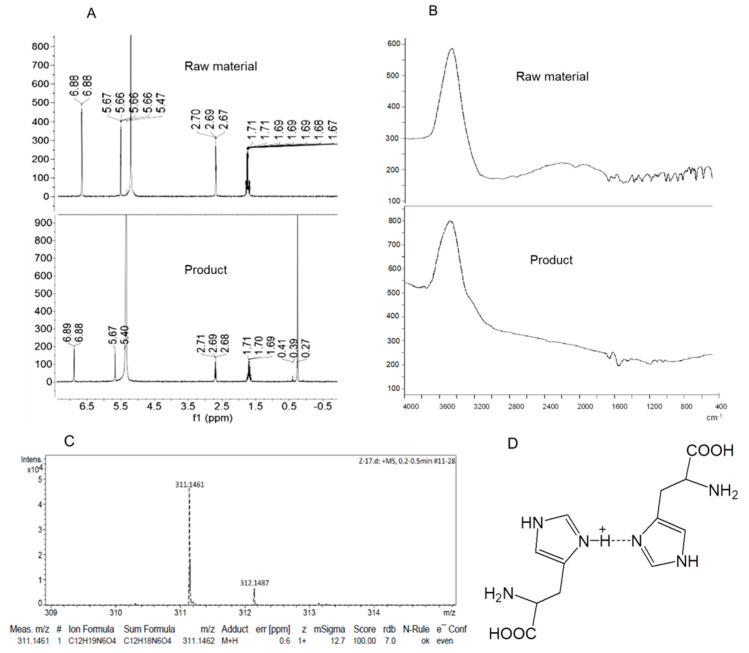
Tructure of H-bihistidine. Comparation of the raw material L-histidine and the product (H-bihistidine) by using ^1^H NMR (**A**) and FTIR (**B**). (**C**) HRMS spectrum of H-bihistidine. (**D**) The chemical structure of H-bihistidine.

The synthesis of H-bihistidine is originally from an unexpected finding. It was found that l-histidine solution reacted with excess ammonium chloride at room temperature, and a new chemical could be precipitated out gradually. The structure of H-bihistidine was, respectively, identified by ^1^H NMR, FTIR and HRMS. The spectrum of ^1^H NMR (400 MHz, deuterium oxide, D_2_O) is shown as follows: δ 6.88 (d, *J* = 1.4 Hz, 1H), 5.67 (s, 1H), 5.40 (s, 6H), 2.71–2.68 (t, *J* = 6.7 Hz, 1H), 1.71–1.69 (m, 2H), 0.27 (s, 3H) (Figure 1A). Compared to the ^1^H NMR spectrum of histidine (raw material), a new peak at 0.27 appears in the spectrum of H-bihistidine (product) (Figure 1A). The FTIR spectra show that a hydrogen bond (about 3200–3000 cm^−1^) displays in the structure of H-bihistidine (Figure 1B). In addition, the HRMS spectrum identifies that the compound is a proton-containing structure (Figure 1C), and the molecular formula is C_12_H_19_N_6_O_4_. Collectively, H-bihistidine is composed of two histidines where the N-1 atom of the imidazole group in histidine and N-1 atom of the other histidine imidazole group are connected by a hemi-protonated intramolecular hydrogen bond (N–H^+^···N) (Figure 1D). The compound remains stable at experimental pH (3–9) and temperature (−20–100 °C).

### 3.2. H-Bihistidine Scavenged H_2_O_2_ and Reduced Cell Injury Induced by H_2_O_2_

**Figure 2:**
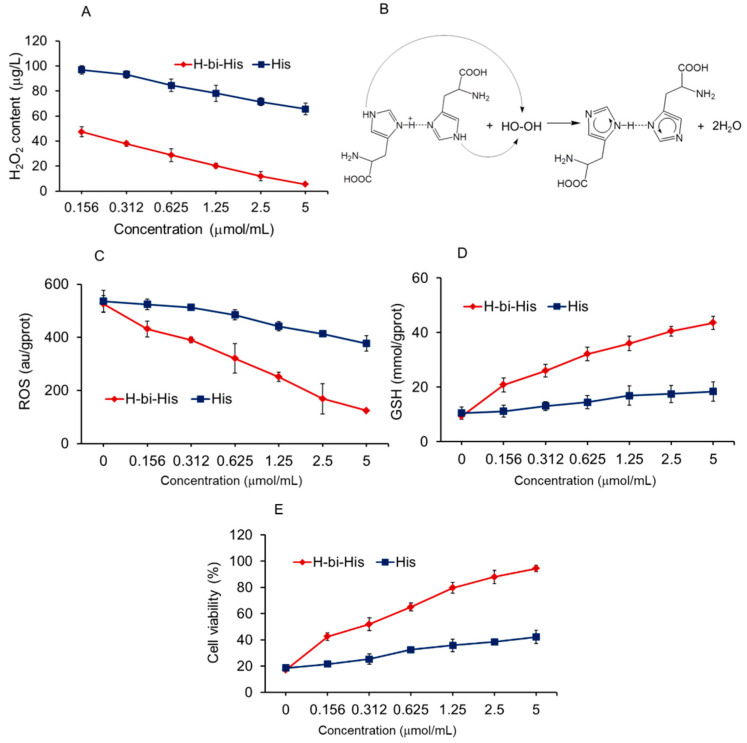
H-bihistidine reduced H_2_O_2_ levels in test tubes and cultured hepatocytes in concentration-dependent manners. (**A**) H-bihistidine decomposed H_2_O_2_ in test tubes. Moreover, H-bihistidine decreased H_2_O_2_ level (**B**) and intracellular ROS content (**C**) in hepatocytes. (**D**) GSH content after H-bihistidine addition in the H_2_O_2_-treated cells. (**E**) H-bihistidine recovered cell viabilities that were reduced by H_2_O_2_. Cells were incubated with 3 mM H_2_O_2_ for 2 h, then H-bihistidine was added into the media. Data were expressed as mean ± SD (*n* = 6 in each concentration).

When H-bihistidine was added to H_2_O_2_ solution in test tubes, it directly decomposed H_2_O_2_ to produce water (Figure 2A,B), indicating that H-bihistidine could be used as a nonenzymatic H_2_O_2_ scavenger. In H_2_O_2_-induced hepatocyte injury, H-bihistidine could reduce ROS concentration (Figure 2C), increase GSH content (Figure 2D), and restore cell viability in concentration-dependent behaviors (Figure 2E). The results demonstrate that H-bihistidine may have a strong anti-free radical capacity.

### 3.3. H-Bihistidine Prevented Palmitate-Induced Cell Injury

**Figure 3:**
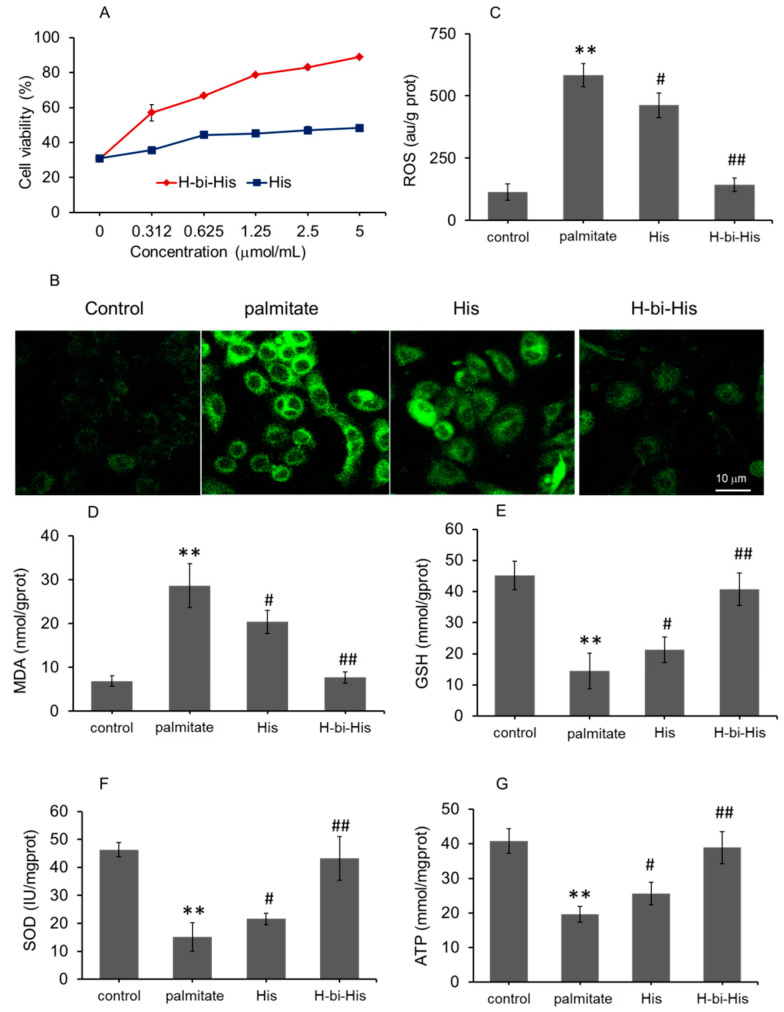
H-bihistidine rescued hepatocytes that were damaged by fatty acid. (**A**) H-bihistidine could increase cell viability in a concentration-dependent pattern. (**B**) DCFH-DA staining showed that ROS level decreased after H-bihistidine addition. Biochemical assay also indicated that H-bihistidine reduced the contents of ROS (**C**) and MDA (**D**) in the hepatocytes that were treated by palmitate. Moreover, levels of GSH (**E**), SOD (**F**), and ATP (**G**) elevated after H-bihistidine addition into the cell media. *n* = 6 in each group. ** *p* < 0.01 compared with the normal control; ^#^ *p* < 0.05, ^##^ *p* < 0.01 compared with the model control.

Palmitate is a saturated fatty acid that is commonly used to simulate the pathogenesis of NAFL in vitro. Here, we established the palmitate-induced hepatocyte injury model to examine the effect of H-bihistidine on adipotoxicity. The results showed that cell viability decreased to about 30% after the cells were incubated with palmitate for 24 h, while the viability enhanced in a concentration-dependent manner following H-bihistidine addition into the cell media (Figure 3A). The cell viability increased to 88.9% when the H-bihistidine concentration reached 5 μg/mL, but it only reached 48.3% at the same concentration in the histidine-treated cells (Figure 3A), suggesting that H-bihistidine has a higher efficiency of anti-lipid peroxidation than histidine. In addition, DCFH-DA (a ROS probe) staining exhibited that green fluorescence obviously enhanced in palmitate-treated cells, while the fluorescence became weak after H-bihistidine addition (Figure 3B), indicating that H-bihistidine could reduce intracellular ROS level. In addition, the biochemical assay showed that contents of ROS and MDA (a lipid peroxide product) elevated only in the palmitate-treated cells (model group), but H-bihistidine was able to reverse the ROS level from 583.7 ± 46.3 to 143.5 ± 26.8 au/g protein, and prevent 73.4% of the MDA formation at the concentration of 5 μg/mL (Figure 3C,D), exhibiting solid antioxidant capacity. Moreover, H-bihistidine can increase the endogenous cellular antioxidant (GSH and SOD) levels that were reduced by the palmitate (Figure 3E,F), and, accordingly, ATP production enhanced after H-bihistidine addition (Figure 3G). This result suggests that H-bihistidine has the capability of rescuing hepatocytes from fatty-acid-induced injury.

### 3.4. H-Bihistidine Restored Cell Viability in CCl_4_-Induced Cell Injury

**Figure 4:**
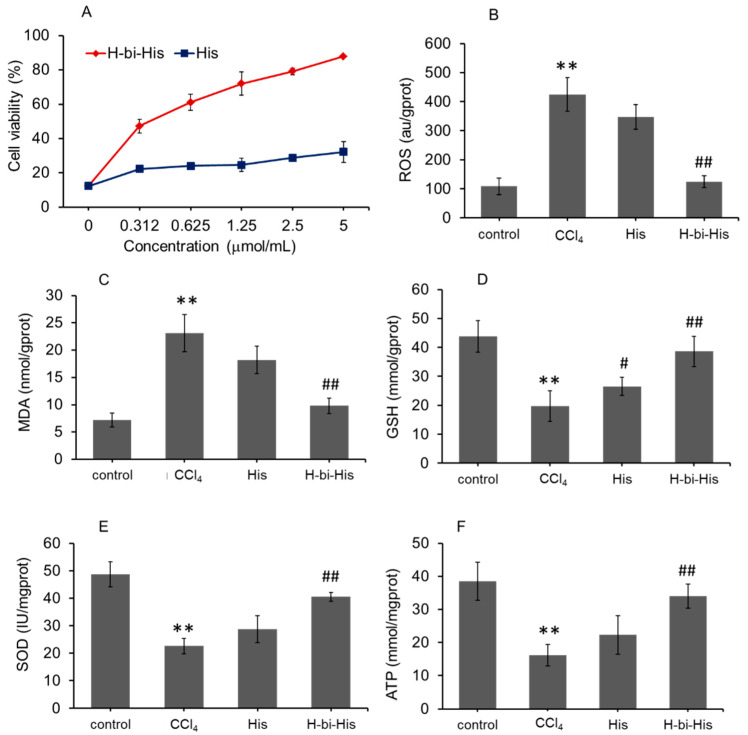
H-bihistidine inhibited CCl_4_-induced hepatocyte injury. (**A**) H-bihistidine increased cell viability in a concentration-dependent manner. The contents of ROS (**B**) and MDA (**C**) decreased, and levels of GSH (**D**), SOD (**E**), and ATP (**F**) elevated after H-bihistidine treatment (*n* = 6). ** *p* < 0.01 compared with the normal control; ^#^ *p* < 0.05, ^##^ *p* < 0.01 compared with the model control.

CCl_4_ processes substantial hepatotoxicity through free radical damage. In the study, 10 mM CCl_4_ incubation for 12 h significantly decreased the cell viability to about 12% (Figure 4A). The contents of ROS and MDA increased (Figure 4B,C) and the levels of antioxidants (SOD and GSH) and ATP reduced (Figure 4D–F). After introducing H-bihistidine into the hepatocyte cell media, cell viability elevated with a concentration-dependent pattern (Figure 4A). The antioxidant level and ATP numbers elevated after H-bihistidine treatment (Figure 4C–F). The results showed that H-bihistidine could enhance cell viability by eliminating free radicals and enhancing energy production.

### 3.5. H-Bihistidine Reduced Serum Transaminase and Lipid In Vivo

**Figure 5:**
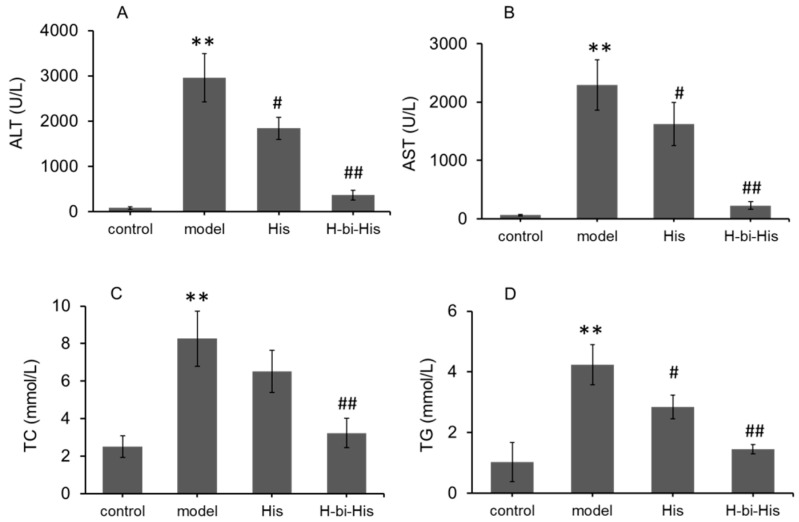
H-bihistidine reduced serum transaminase and lipid levels in the model mice of NAFLI. (**A**) ALT activity. (**B**) AST activity. (**C**) TC level. (**D**) TG level. *n* = 10 in each group. ** *p* < 0.01 compared with the normal control; ^#^ *p* < 0.05, ^##^ *p* < 0.01 compared with the model control.

After the mice with NAFL acquired 5 mg/kg of H-bihistidine treatment for 7 consecutive days, the sera were obtained for transaminase activity and lipid content calculation. The results revealed that the ALT and AST levels were significantly elevated in the fatty liver mice (Figure 5A,B), suggesting that the high-fat-diet-induced liver injury in mice was in agreement with a previous report [16]. After H-bihistidine administration, transaminase activities decreased close to the control levels (Figure 5A,B). Moreover, serum TC and TG levels elevated after the mice were fed with the high-fat and high-cholesterol diets (*p* < 0.01) (Figure 5C,D). Moreover, the levels of TC and TG retarded significantly in the H-bihistidine-treated group in comparison with the fatty liver mice (*p* < 0.01) and nearly reached the control levels (Figure 5C,D), suggesting that H-bihistidine could reduce cell injury from lipid metabolism.

### 3.6. Treatment of Mouse Fatty Liver with H-Bihistidine

**Figure 6:**
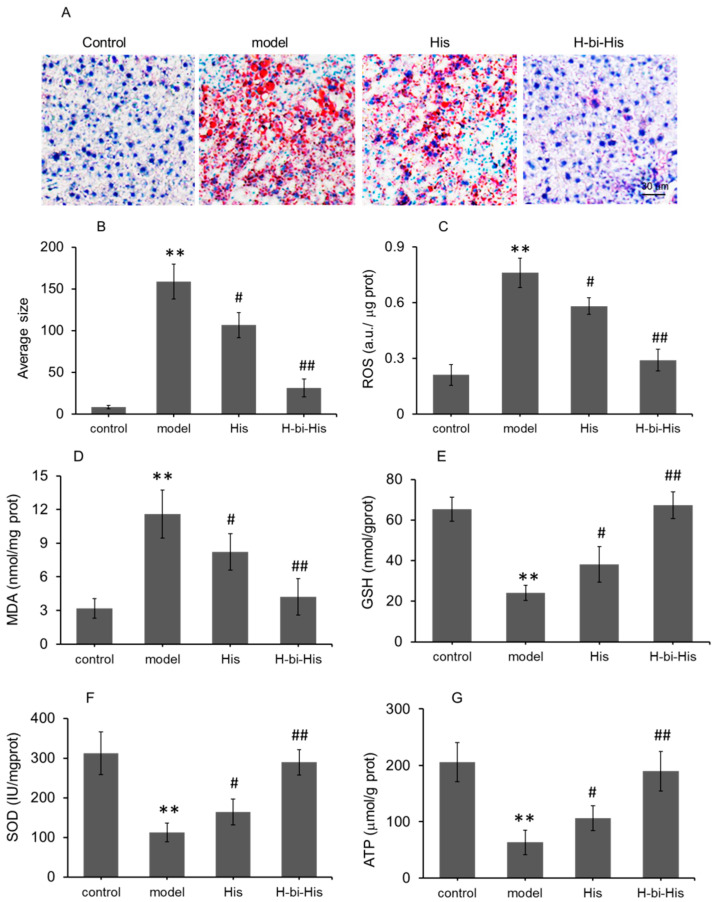
H-bihistidine inhibited liver cell injury in the mice treated with high-fat diet. (**A**) Liver sections were stained with Oil Red O. (**B**) Average size of accumulation of lipid droplets in the liver sections. (**C**) ROS content. (**D**) MDA level. (**E**) GSH level. (**F**) SOD activity. (**G**) ATP content. *n* = 10 in each group. ** *p* < 0.01 compared with the normal control; ^#^ *p* < 0.05, ^##^ *p* < 0.01 compared with the model control.

The staining with Oil Red O revealed a persistent fat deposit in the mouse liver after the mice were fed with the high-fat diet for about 8 weeks (Figure 6A), whereas the lipid droplets were prominently retarded after H-bihistidine administration (Figure 6A,B). To evaluate the effects of H-bihistidine on the high-fat-diet-produced liver injury, levels of ROS, MDA, GSH, and activity of SOD in mouse liver homogenates were calculated, respectively. The results revealed that, after mice were given the high-fat diets, the hepatic ROS and MDA levels were prominently elevated (Figure 6C,D), and cellular antioxidant GSH content and SOD activity remarkably decreased (Figure 6E,F). However, after H-bihistidine administration, both ROS and MDA decreased compared to the fatty liver mice; meanwhile, GSH content and SOD activity elevated, respectively (*p* < 0.01). Moreover, there was no apparent difference in ROS, MDA, GSH, and SOD levels between the normal control group and the H-bihistidine-treated group (Figure 6C–F). Accordingly, ATP content in the liver homogenate of the fatty liver mice was prominently reduced in comparison with the control mice (Figure 6G), while H-bihistidine produced a 66.7% increase in ATP level in the H-bihistidine (Figure 6G). Moreover, no obvious difference was revealed in the ATP level between the control group and H-bihistidine-treated mice. Compared with H-bihistidine, histidine exhibited a relatively weak effect against liver injury, despite statistical differences in the biochemical indexes related to the model mice. The results stated that the H-bihistidine could rescue the hepatocytes from free radical injury.

### 3.7. H-Bihistidine Prevented CCl_4_-Induced Liver Injury

**Figure 7:**
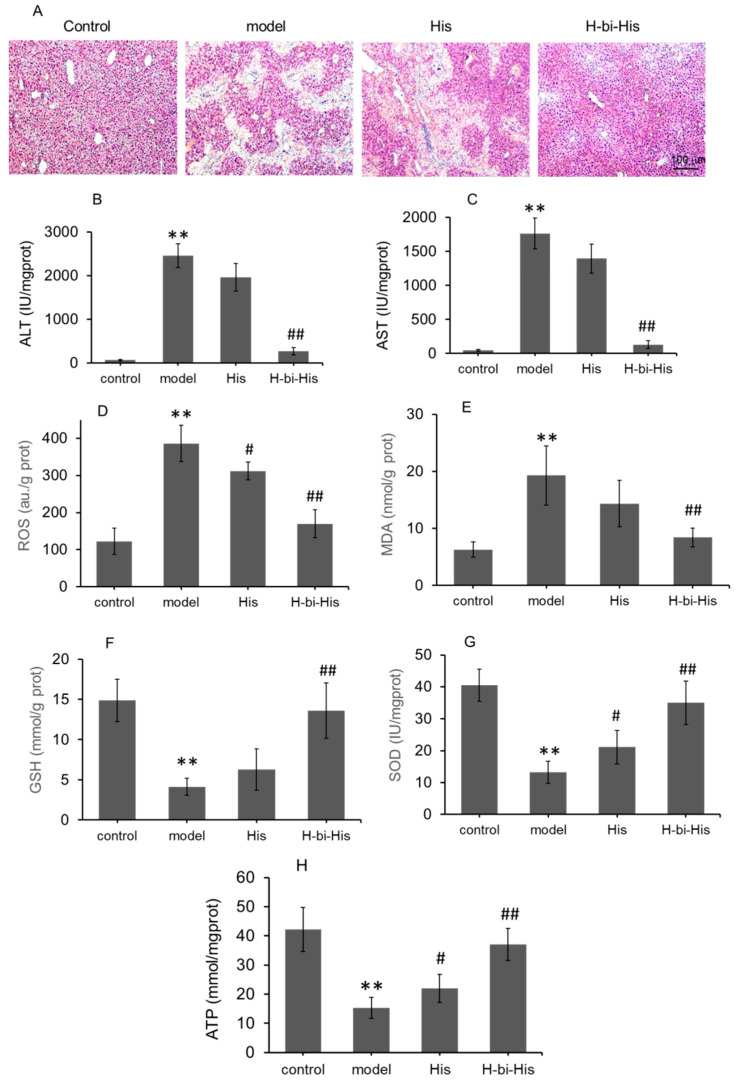
Therapeutic effects of H-bihistidine on liver injury induced by CCl_4_. (**A**) HE staining of liver sections. The contents of ALT (**B**) and AST (**C**) were assayed in mouse serum. (**D**) ROS content. (**E**) MDA level. (**F**) GSH level. (**G**) SOD activity. (**H**) ATP content. *n* = 10 in each group. ** *p* < 0.01 compared with the normal control; ^#^ *p* < 0.05, ^##^ *p* < 0.01 compared with the model control.

When CCl_4_ is subcutaneously injected into mice, it can produce hepatocellular injury, which is widely utilized to prepare animal models of liver injury. On the sections of HE staining, pathological changes appeared in liver tissue of only CCl_4_-treated mice (model group), including swelling and necrosis, and even disappearance of hepatocytes in areas of necrosis around central veins (Figure 7A). Compared to the model group, the liver tissues of the histidine-treated group were slightly improved, and there were hepatocytes in the injured area. However, H-bihistidine significantly improved the liver tissue morphology and the necrosis area disappeared, indicating that H-bihistidine could inhibit the liver injury induced by CCl_4_ and promote the recovery of the hepatocytes (Figure 7A). In addition, the elevated serum ALT and AST levels in CCl_4_-poisoned mice reduced after H-bihistidine treatment (Figure 7B,C), suggesting the therapeutic effect of H-bihistidine against CCl_4_-induced free radical injury and hepatocyte degeneration in the liver tissue.

Moreover, the anti-free radical effect of H-bihistidine is further verified by biochemical measurement, as shown in Figure 7D–H. After H-bihistidine administration, contents of ROS and MDA significantly reduced (Figure 7D,E), and levels of GSH, SOD, and ATP increased (Figure 7F–H), indicating that the H-bihistidine could scavenge the free radical injury induced by CCl_4_. However, histidine displayed insufficient anti-free radical capacity in the liver injury as compared with H-bihistidine.

### 3.8. H-Bihistidine Prevented Liver Fibrosis Induced by Both High-Fat Diet and CCl_4_

**Figure 8:**
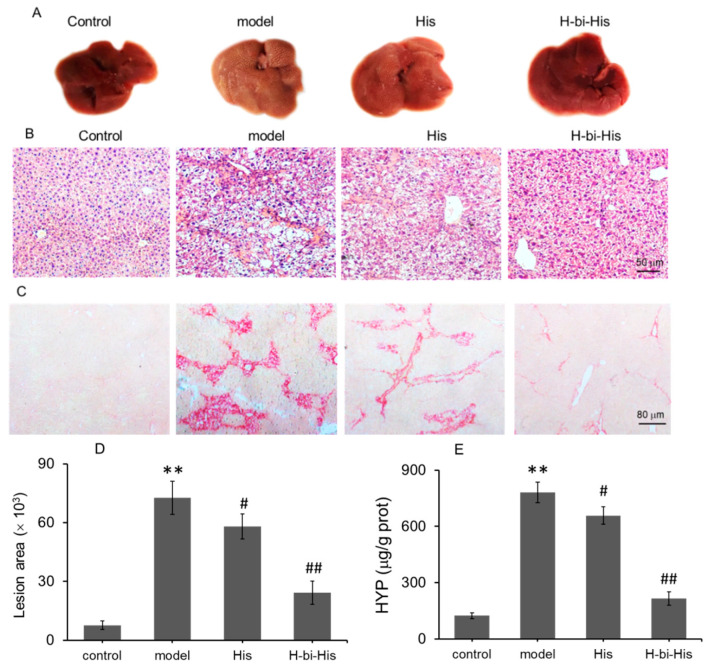
H-bihistidine alleviated liver fibrosis induced by high-fat diet and CCl_4_ administration in mice. (**A**) Liver morphology in each group. (**B**) HE staining. (**C**) Sirius red staining. (**D**) Fibrotic plaque size. (**E**) HYP content. *n* = 10 in each group. ** *p* < 0.01 compared with the normal control; ^#^ *p* < 0.05, ^##^ *p* < 0.01 compared with the model control.

CCl_4_ injections with high-fat diet can cause severe free radical injury, resulting in the progress of fibrosis in mice. Here, we used the animal model of liver fibrosis produced by both high-fat diet and CCl_4_ administration to examine the anti-fibrosis activity of the potential therapeutic agent. As shown in Figure 8A, the healthy liver showed a reddish-brown color with a smooth surface, but the fibrotic liver appeared light brown with white punctate nodules on the surface. Histidine therapy could reduce the number of the nodules on the liver surface and partly improve the morphology of local liver areas, while H-bihistidine almost recovered the liver morphology to normal. H-bihistidine shrunk the fibrotic area and recovered the liver structure (Figure 8B–D). In addition, the biochemical measurement of hydroxyproline (HYP; an indicator of fibrosis) level suggested that the HYP was significantly inhibited in the H-bihistidine-treated mice (Figure 8E).

## 4. Discussion

**Figure 9:**
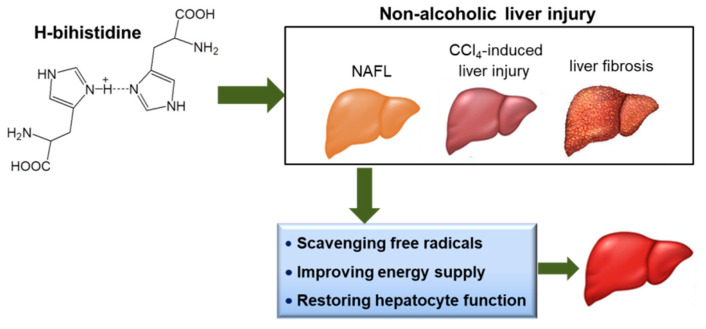
Schematic diagram of H-bihistidine in the treatment of nonalcoholic liver injury (NLI). H-bihistidine can remedy NLI (including NAFL, CC1_4_-induced liver injury, liver fibrosis) through scavenging free radicals and increasing ATP production.

Although NLI has been a common disease worldwide, there is still a lack of therapeutic agents. Elimination of predisposing factors may be the best option for the disease, but pharmacological intervention is required in most cases. Thus, effective candidates should be exploited to inhibit or reverse hepatocyte injury. This study explores the anti-free radical capability of the protonated compound H-bihistidine and proves that it could be used as a new therapeutic candidate for treating NLI (Figure 9).

Histidine is an essential amino acid containing the imidazole group. Histidine is usually located in the active center of oxidoreductase and plays an essential role in gaining and losing protons of the imidazole group. Nevertheless, with histidine alone, it is hard to lose the proton of the imidazole group, which exerts antioxidant effect only through metal binding or conversion to carnosine [17]. For example, histidine inhibits lipid peroxidation by forming a complex with ferric iron, and then prevents ferrous iron generation and Fenton reaction [18]. However, when histidine conjugates with other molecules (such as alanine), the proton of the imidazole group of the product is easy to lose, resulting in a strong antioxidant effect [19]. Here, we synthesize dimer histidine compound through protonation technology and find that the hydrogenated bihistidine efficiently provides their protons of the imidazole group to neutralize free radicals, showing direct and solid anti-free radical effect. When the H-bihistidine is directly added into the H_2_O_2_ solution, protons on imidazole groups of H-bihistidine can separate HO–OH to form H_2_O, and H-bihistidine molecule is stabilized by resonance (Figure 2B). In the cells treated with H_2_O_2_, H_2_O_2_ generates OH radicals (^•^OH) under the action of peroxidase. It is known that ^•^OH is one of the most vital free radicals and can severely damage biomembrane and protein in cells, so the elimination of ^•^OH can protect cells against cell rupture [20]. Since H-bihistidine can donate its protons of imidazole groups to ^•^OH, the protons neutralize ^•^OH to form H_2_O, eliminating cell damage caused by H_2_O_2_.

In the case of NLI, an inevitable cause is lipid peroxidation (LP), due to the detachment of hydrogen from fatty acid to form ROO^•^ that can initiate a complex cascade reaction until aldehyde formation (such as MDA) [21,22]. Thus, it is crucial to maintain redox balance with antioxidants during NLI, since they can prevent the deleterious effects of LP. In this study, H-bihistidine can affect cellular antioxidant balance through its anti-free radical capacity. In this regard, H-bihistidine could donate its proton to ROO^•^, then eventually reduce the MDA level. In addition, H-bihistidine can enhance the cellular antioxidant system, including GSH and SOD, which will produce a beneficial effect to decrease cell oxidative injury and restore cell function. In the animal model of NAFL, high-fat diet can produce many free radicals in the liver, since the liver is the main organ of lipid metabolism in mammals. The free radicals can damage mitochondrial structure and function, leading to lipid accumulation in the liver [23]. However, H-bihistidine can scavenge the free radicals and then recover mitochondrial function, evaluated by reduced lipid accumulation and elevated ATP levels. These effects can lead to the recovery of hepatocyte function, thereby decreasing lipids, AST, and ALT in blood.

Besides high-fat diets, NLI can be initiated by toxins. CCl_4_ is widely used among the toxins to induce liver damage by forming trichloromethyl radical (CCl_3_^•^) and, subsequently, trichloromethyl peroxy radical (CCl_3_OO^•^) [24]. The species are highly reactive and can attack lipids and proteins, resulting in cell membrane rupture [25]. In this case, histidine displays inadequate antioxidant capacity and cannot significantly prevent CCl_4_-induced liver injury, while H-bihistidine exhibits vigorous anti-free radical activity, evaluated by the elevation of the antioxidant system (such as SOD and GSH) and energy production.

NLI induced by free radical damage can continue to develop into liver fibrosis, and then a progression of cirrhosis and carcinoma. To determine whether H-bihistidine can retard the process of liver fibrosis through its antioxidant activity, the animal model of liver fibrosis induced by CCl_4_ and high-fat diet is used in the study. The H-bihistidine shows a highly hepatoprotective effect and prevents fibrosis. The effects of H-bihistidine in liver fibrosis may be closely associated with the inhibition of downstream inflammation and transdifferentiation of hepatic stellate cells, followed by free radical damage, leading to reduced collagen synthesis. The positive effects of H-bihistidine in different protocols of NLI suggest that H-bihistidine has the hepatoprotective capacity as a potent free radical scavenger.

## 5. Conclusions

In summary, here we synthesized the protonated compound H-bihistidine with intense anti-free radical activity, which could be used to combat the free-radical-induced cell injury. Due to the molecular structure, H-bihistidine possesses directly anti-free radical properties and is considered safe for animals. The effect of H-bihistidine has been identified in several liver damage models by using hepatocyte damage agents, such as CCl_4_, high-fat diet, and both. H-bihistidine displays an excellent hepatoprotective activity based on its anti-free radical capacity. The other beneficial effects of H-bihistidine, including inflammatory inhibition and tissue repair, will be further revealed. Nevertheless, the study would provide a new therapeutic option for NLI and the potential for free-radical-induced diseases.

## Data Availability

The data presented in this study are available in this manuscript.
